# Electrically and Geometrically Tunable Photon Pair Entanglement from Ferroelectric Nematic Liquid Crystal

**DOI:** 10.1002/advs.202515206

**Published:** 2025-10-24

**Authors:** Sara Klopčič, Aljaž Kavčič, Nerea Sebastián, Matjaž Humar

**Affiliations:** ^1^ Jožef Stefan Institute Ljubljana 1000 Slovenia; ^2^ Faculty of Mathematics and Physics University of Ljubljana Ljubljana 1000 Slovenia; ^3^ CENN Nanocenter Ljubljana 1000 Slovenia

**Keywords:** ferroelectric nematic liquid crystals, photon entanglement, spontaneous parametric down‐conversion, tunability

## Abstract

Entangled photons are a cornerstone of quantum technologies, enabling applications from secure communication to quantum computing. A longstanding challenge is to develop a compact source that would generate polarization‐entangled photons with tunable quantum state on demand. The promising materials for such sources are ferroelectric nematic liquid crystals (FNLCs), due to their nonlinear optical properties and easily controllable configuration. In this work, it is demonstrated that the polarization state and the degree of entanglement of photon pairs generated within FNLCs can be changed in a controllable and reversible manner. First, tuning of the entanglement is demonstrated via sample geometry with twisted FNLC configurations in a sample of varying thickness. Secondly, by applying an electric field, the degree of entanglement can be tuned in real time. In both scenarios, the degree of entanglement can be adjusted from nearly entirely separate photons to fully entangled. These findings represent a significant step toward tunable quantum sources that can produce any desired polarization state on demand. In the future, by adding more electrodes, different parts of the sample could be controlled individually, allowing for the creation of pixelated quantum light sources.

## Graphical abstract

By exploiting the strong nonlinear optical response and controllable molecular ordering of ferroelectric nematic liquid crystals (FNLCs), these materials can serve as tunable sources of polarization‐entangled photons. Adjusting the sample geometry or applying an external electric field enables continuous control of the entanglement degree, advancing the development of on‐demand, pixelated quantum light sources for emerging quantum technologies.

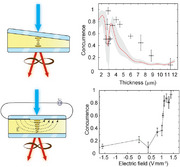

## 1 Introduction

Liquid crystals (LCs) are among the most widely used materials in light modulation and transformation,^[^ ^1^ ^]^ owing to their fluidity, birefringence and high response to external stimuli.^[^ ^2^ ^]^ They are a key ingredient of optical devices such as LC displays, spatial light modulators, variable filters and more.^[^ ^1^, ^3^ ^]^ The latest generation of liquid crystals, the ferroelectric nematic liquid crystals (FNLCs), show spontaneous polarization **P** parallel to the nematic director $\hat{n}\;\equiv \;-\hat{n}$, where the director denotes a unit vector field that specifies the average local orientation of the long molecular axes. While individual molecules are free to fluctuate, the director represents their collective alignment direction that captures their quadrupolar orientational order.^[^ ^4^, ^5^, ^6^, ^7^ ^]^ The lack of inversion symmetry is accompanied, in some cases, by large optical nonlinearity.^[^ ^8^, ^9^, ^10^ ^]^ With it, several new possibilities for optical applications employing LCs have emerged, especially in the realm of highly efficient tunable nonlinear optical devices.^[^ ^11^, ^12^ ^]^

Since spontaneous parametric down‐conversion (SPDC) is the most popular method for generating quantum light and it relies on optically non‐linear materials, FNLCs also open the door for new avenues in the generation of quantum light. In our recent work,^[^ ^13^ ^]^ we have demonstrated for the very first time that photon pairs can be generated within LCs, which is also the first demonstration of SPDC in any organic material. In combination with the already known advantages of LCs in terms of the ease of their manipulation and tunability, we demonstrated that the state of the photon‐pairs is highly tunable and can be altered very easily and quickly by manipulating the orientation of the director in the sample. This can be achieved either by designing the confining liquid crystal cell in a way that the boundaries are forcing a given configuration or actively in real time by applying an electric field. This opens the door to a whole new field of tunable quantum optics. However, so far, only the generation of photon pairs from FNLCs and the control of simple polarization states have been successfully realized. In our previous work,^[^ ^13^ ^]^ we have not achieved complete entanglement of photons, nor have we explored the entanglement dependence on the liquid crystal cell properties, such as boundary conditions and thickness. Although the electric field was used to switch between simple states, it was not used to vary the degree of entanglement in a controllable manner. Realizing this could enable further applications of FNLCs as quantum light sources.

When engineering the states of photon‐pairs through SPDC, the goal is usually to create entangled photon pairs.^[^ ^14^, ^15^, ^16^, ^17^ ^]^ This property can be harnessed in a variety of different quantum technologies, ranging from quantum cryptography, quantum key distribution protocols, quantum communications, quantum metrology and others.^[^ ^18^, ^19^, ^20^, ^21^, ^22^ ^]^ However, for certain applications, such as multipixel tunable quantum devices—a high‐dimensional multiphoton quantum source^[^ ^23^ ^]^—it would be necessary to be able to vary the degree of entanglement in space and time controllably. Moreover, tunability provides control not only over entanglement but also over classical properties such as emission intensity and polarization, greatly extending the versatility of the source. This flexibility enables balancing between brightness and entanglement depending on the application requirements, while the ability to tune polarization independently further extends its utility. Importantly, even partially entangled states are valuable for many quantum tasks, including Bell tests, teleportation, and quantum key distribution.^[^ ^24^ ^]^ Unfortunately, solid‐state crystals do not allow for simple changes in their structure to control the state of the generated photons and, consequently, the degree of entanglement, as the rigid crystal lattice can hardly be altered, especially in a reversible manner. While platforms such as thin films, metasurfaces, and waveguides offer limited forms of tunability, they typically suffer from very low brightness and rely on externally imposed conditions rather than material‐intrinsic control.^[^ ^25^, ^26^, ^27^, ^28^ ^]^ Thin 2D materials yield pair rates far below 1 Hz and rely on modulating the pump beam, while metasurface‐based sources remain weak (<10Hz at 100 mW) and require resonant pump‐wavelength matching that makes them narrowband and fabrication‐dependent. In contrast, FNLCs combine significantly higher photon‐pair brightness with intrinsic tunability that originates directly from reconfiguration of the liquid‐crystal structure under external fields. This intrinsic reconfigurability allows continuous adjustment of entanglement, polarization, and intensity without modifying pump conditions or selective detection. For these reasons, FNLCs could enable locally selective, pixelated, or multiplexed sources in the future.

In this study, we demonstrate that a high degree of polarization entanglement can be achieved in FNLCs through simple tuning of the material properties. Furthermore, we demonstrate how our samples enable tuning within the entire range of entanglement, from completely separate photons to completely entangled ones, by setting the correct parameters. We show that entanglement strongly depends on the combination of the director twist and thickness of the sample, as well as the direction and strength of the electric field applied to it. Lastly, the demonstrated tunability of the material simultaneously enables control over classical properties such as emission intensity and polarization, which offers flexibility across a wide range of quantum and classical applications.

## 2 Results and Discussion

**Figure 1:**
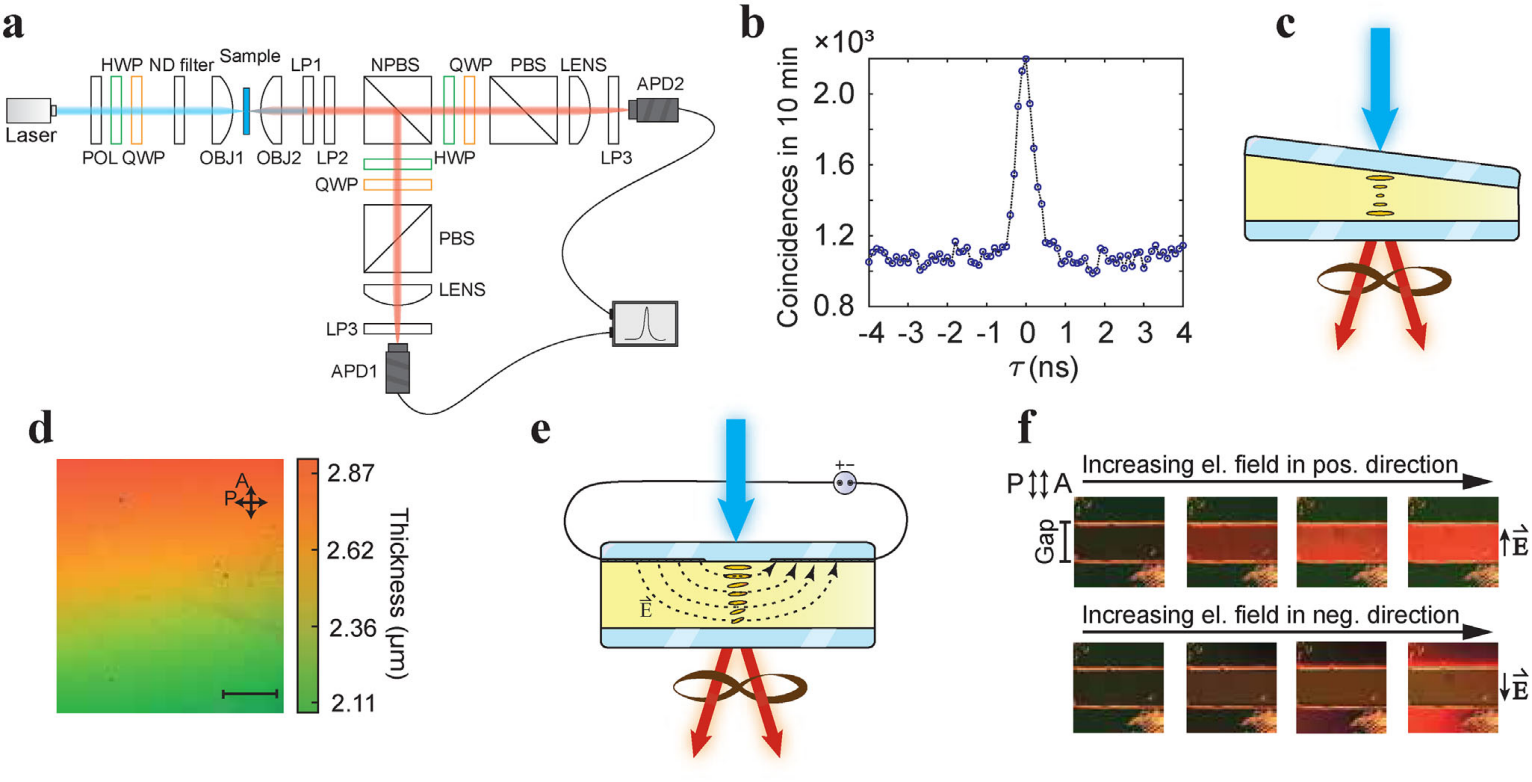
FNLC sample configuration and measurement of the photons generated through SPDC. a) Experimental setup for generation and detection of photon pairs from FNLC. Hanbury Brown ‐ Twiss setup is used for polarization tomography. HWP, half‐wave plate; QWP, quarter‐wave plate; ND filter, neutral density filter; OBJ, objective; LP, long‐pass filter; NPBS, non‐polarizing beam‐splitter; PBS, polarizing beam‐splitter; APD, avalanche photodiode detector. b) A typical histogram of time delays between two consecutively detected events on detectors. The peak at delay zero corresponds to the simultaneous generation of photon pairs. c) A schematic representation of the wedge FNLC sample producing entangled photon pairs. A wedge liquid crystal cell with varying thickness is composed of two rubbed glass plates and the FNLC sample in between. The pump beam enters the cell through the top glass. d) Polarizing optical microscopy image of one part of the wedge cell between crossed polarizers. The director on the top surface is parallel to the direction of the wedge and rotates for 180° with the thickness of the cell. Different colors are the result of different sample thicknesses. Scale bar, 500 µm. e) A schematic representation of the FNLC sample with electrodes and the electric field between them. Thickness and electrode gap are not to scale. f) Polarization microscopy of the sample with 90° director twist between parallel polarizer and analyzer at 0, 0.2, 1, and 1.4 V mm^−1^ in the gap region. The changing color is the result of different director configurations when applying an electric field in plus (upper row) and minus (bottom row) directions. The width of the gap is 500 µm.

All our experiments were performed with the room‐temperature ferroelectric nematic material FNLC‐1751. The only significant component of the nonlinear susceptibility tensor *d* is *d*_33_ which has a value of approximately 20 pm V^−1^.^[^ ^13^ ^]^ For comparison, commonly used nonlinear crystals span a wide range of second‐order nonlinear susceptibilities, from less than 0.5 pm V^−1^ in KDP and about 2 pm V^−1^ in BBO, to 25–35 pm V^−1^ for LiNbO_3_, and exceeding 100 pm V^−1^ in materials such as GaAs.^[^ ^29^, ^30^ ^]^ The liquid crystal sample is assembled in a cell consisting of two glass plates with liquid crystal film between them. Unidirectional rubbing of polyimide‐coated glass substrates induces parallel surface alignment of the liquid crystal molecules, with the polarization vector **P** oriented opposite to the rubbing direction.^[^ ^31^, ^32^, ^33^ ^]^ This method enables the fabrication of liquid crystal cells with controlled variations in the director twist across the cell thickness by adjusting the relative orientation of the rubbing directions on the top and bottom surfaces. The FNLC is pumped using a continuous‐wave (CW) laser at 450 nm and power of 1 mW (**Figure** **1**a) and the generated SPDC signal is captured with single photon avalanche photodiodes (APDs). At this power, the laser did not induce any temporary or permanent damage or reorientation of the LC. The typical single count rate on a single detector when the laser is turned on, is around 100 kHz ± 20 kHz, which is primarily due to the fluorescence generated in the sample and optical elements, and to a smaller extent due to the weak surrounding light. Because the photon pair count rate from SPDC is more than 1000 × weaker than the background, we cannot detect it directly. Instead, we measured the SPDC signal using a standard Hanbury‐Brown and Twiss (HBT) interferometry setup, which yields a peak in the histogram of time delays (τ) between two consecutively detected events (coincidences) on the detectors at τ = 0 (Figure 1b). In our measurements, coincidence rates are typically in the 1–30 Hz range with coincidence to accidental ratio (CAR) values around 1–4, limited mainly by significant fluorescence at short detection wavelengths and restrictions of the current detection system. With increasing power, the background grows faster than the coincidence values. This leads to a decrease in CAR with higher pumping power. In addition, at higher powers, also higher orders of nonlinear processes can occur. For these reasons, we found the power of 1 mW to be optimal. In our previous work with the same material and an optimized detection system at telecom wavelengths, we demonstrated much higher performance with CAR values exceeding 15.^[^ ^13^ ^]^ Our previous study^[^ ^13^ ^]^ demonstrated that the spectrum of the generated photon pairs is broadband. However, it is constrained by the cut‐off wavelengths of the employed 840 nm long‐pass filter. In the present setup, the full width at half maximum (FWHM) of the coincidence peak is primarily determined by the timing jitter of the avalanche photodiodes (APDs) and associated electronics, which is approximately 350 ps and thus constitutes the dominant contribution to the observed peak width. We were interested in the polarization state of the generated photon pair, described with a two‐photon wave function, which is represented as a Fock state

(1) $$\left|\psi \right\rangle =c_{1}{\;|2\rangle }_{H}{\left|0\right\rangle }_{V}+c_{2}{\;|1\rangle }_{H}{\left|1\right\rangle }_{V}+c_{3}{\;|0\rangle }_{H}{\left|2\right\rangle }_{V}\;,$$

where *H* stands for horizontally polarized photon state, *V* vertical polarized photon state and *c*_1_, *c*_2_ and *c*_3_ are complex amplitudes. Since the photons are distinguished only by their polarization and there is no other degree of freedom, the state remains the same if we interchange the polarizations of the two photons. Therefore, states |*H*〉|*V*〉 and |*V*〉|*H*〉 are equivalent and can be described with the Fock state |1〉_*H*_|1〉_*V*_. This means there are only three base states ‐ qutrits ‐ instead of four.^[^ ^34^ ^]^ Separating the |*H*〉|*V*〉 and |*V*〉|*H*〉 components would require access to additional degrees of freedom beyond polarization, such as spatial mode filtering. While those Bell states, that contain |*H*〉|*V*〉 and |*V*〉|*H*〉, could not be observed on our current setup, Bell states of type |*H*〉|*H*〉 ± |*V*〉|*V*〉 can, in principle, be realized under suitable conditions in our system (although that was not the goal of our study and remains one of the possibilities for future work).

For describing our state we use a 3 × 3 density matrix to maintain a general formalism as well as to quantify the state purity. We have observed that the detected photons are, to an excellent approximation, in pure polarization states. We used the so‐called polarization tomography method to reconstruct the density matrix from the measurements. This method involves measuring nine different combinations of photon pair polarizations, as described in detail in Experimental Section.

One of the key points of interest is the degree of entanglement, which (for pure states) is characterized as concurrence

(2) $$C=|2c_{1}c_{3}-c_{2}^{2}|\;.$$

Photon pairs that are fully entangled in polarization are characterized by *C* = 1. On the other hand, completely separable (non‐entangled) photons have *C* = 0.

Due to the predominant *d*_33_ nonlinear tensor component, the nature of SPDC generation in a sample without a twist is type‐0. However, in case of a twist, SPDC generation remains type‐0 only locally, while effectively we can not categorize it with a certain type. Different director orientations with respect to the pump beam polarization lead to locally different *d*‐tensor from the rest of the cell and thus influence the polarization state of the generated photon pairs. Moreover, the polarization of the pump photons as well as the generated photons transforms during their propagation due to the optical anisotropy and orientation of the material. This means that different thicknesses with the same director twist, as well as different twist structures within the same thickness, would lead to different photon states. Consequently, the degree of entanglement also depends on these two parameters, which we can experimentally manipulate.

In our case, we designed two systems. First of all, to study the effect of the sample's director twist and thickness, we have constructed a 180°‐twist wedge cell, where antiparallel rubbing on both glass plates ensures that the director twist is 180° in all positions and where thickness linearly increases from one end to the other (Figure 1c). Unless stated otherwise, we will always assume that the pump beam enters the cell through the top glass and exits it at the bottom. In between, it is influenced by the liquid crystal material. The varying thickness in combination with the material's birefringence contributes to the change in color along the wedge direction, when observing the sample between crossed polarizers (Figure 1d).

Moreover, the effect of an external electric field on the director orientation and, therefore, on the generated photon pairs, is studied. In this case, cells with a constant thickness and different boundary conditions are used (Figure 1e). An approximately homogeneous electric field region is generated in the 500 µm wide gap between the electrodes, where field strength variation with distance from the surface is negligible due to the small (∼5μm) thickness of the cell. Due to the linear coupling of the sample polarization and the applied electric field, the ground structure can be easily deformed into a field‐induced equilibrium structure determined by the direction of the electric field and the boundary conditions. This is visible from the polarizing optical microscopy images (Figure 1f), where the transmission color changes evidence the reorientation of the director under varying electric field.

### 2.1 Entanglement Control via Twist and Thickness

**Figure 2:**
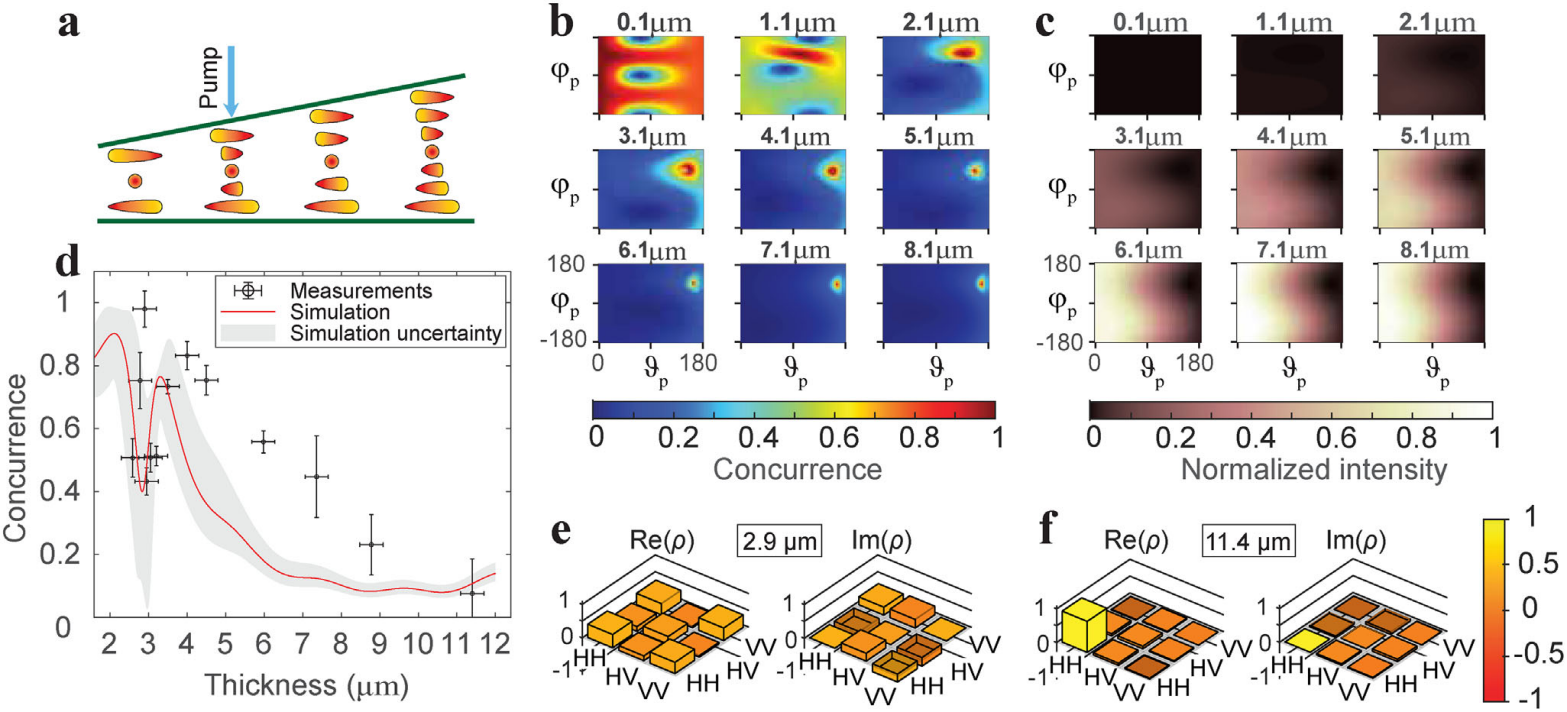
Thickness dependence of concurrence and intensity of the generated photon pairs. a) Wedge cell with anti‐parallel rubbed surfaces, which induce 180° director twist along the thickness of the cell. b) Simulated concurrence for the left‐handed structure depicted in (a), based on Jones calculus for calculating concurrence at different thicknesses of the sample for different pump polarizations. Polarization is defined with angles from the Jones vector ϑ_*p*_ and φ_*p*_. The range of both angles spans all possible elliptical polarizations, including linear and circular ones. c) The results of the simulation for the normalized intensity of the same generated photon pairs as in (b). d) Measured values of concurrence at different sample thicknesses (black points) and simulated concurrence with the same parameters as in experiments (red curve). Grey area represents calculated values for angles between ± 5° from central angles ϑ_*p*_ and φ_*p*_. e,f) Real and imaginary parts of the density matrix for generated photon pair states at 2.9 µm and 11.4 µm, respectively.

The thickness of our wedge cell with 180° director twist ranged from 2.5 µm to 20 °m (**Figure** **2**a). Due to the symmetry of this particular configuration, both clockwise and anti‐clockwise twisted domains are possible within the same sample, as both are energetically equivalent. Extended domains with a given handedness of rotation are formed, separated by well‐defined domain walls. This feature is not critical for the present work, as measurements can always be performed selectively within a single domain, and apart from handedness, the generated quantum states are otherwise identical. In particular, we performed the measurements in the left‐handed domain. In terms of polarization of pump photons, the direction parallel to rubbing is defined as horizontal and the direction perpendicular to it in the sample plane as vertical. Before the measurements, simulations were run to predict the optimal thickness and polarization of the pump beam to achieve highly entangled photon pairs. Simulations developed in our previous paper^[^ ^13^ ^]^ are based on a Jones‐matrix formalism and SPDC theory to compute the resulting two‐photon polarization state from the LC cell parameters and pump polarization. More details can be found in the Experimental Section. To explore the available parameter space of our samples configured as cells in Figure 2a, we sweep across a dense grid of pump polarizations **e**_*p*_ parametrized by angles ϑ_*p*_ and φ_*p*_

(3) $$e_{p}=\left[\begin{array}{c} \cos\frac{\vartheta_{p}}{2}\\ \sin\frac{\vartheta_{p}}{2}e^{i\varphi_{p}} \end{array}\right]$$

and repeat the calculations for a range of cell thicknesses. The results are presented in Figure 2b as 2D maps of concurrence versus the two pump angles where each subfigure corresponds to one thickness. These maps show, for any given thickness, which pump polarizations produce high entanglement (red regions) and which produce little or no entanglement (blue). It is clear that regions of high entanglement are much larger at smaller thicknesses, which means that it is easier to produce entangled states in the thinner region of the sample as opposed to the thicker regions, where entanglement can only be produced for a very limited range of pump polarizations. The sensitivity of entanglement to pump polarization and thickness arises because the contributions from different positions along the sample must interfere in exactly the right manner to produce a high degree of entanglement. Since the optical path length varies with thickness, the relative phase accumulated by different contributions changes, shifting and restraining the optimal pump polarization that balances these contributions. Thicker samples reduce the likelihood that all contributions will coherently interfere to produce a highly entangled state, so entanglement is more sensitive to thickness than in standard SPDC crystals. While thinner samples are beneficial in this respect, they produce photon pairs at a much lower rate (Figure 2c). The intensity at thicknesses below 2 µm can be smaller by a factor of 10 than the intensity above 7 µm. As the thickness increases, the incoming light polarization required to achieve high entanglement begins to approach vertical polarization. That is not in favor of high intensity, because the director is oriented in the horizontal direction at the top and, thus, the large component of the *d*‐tensor is experienced only by horizontally polarized light. Taking into account both aspects, we searched for highly entangled photon states at around 3 µm, where the intensity due to thickness is already a bit higher and the high entanglement region is still large enough for us to be able to find the right pump polarization.

To generate maximally entangled states at this thickness with sufficient intensity, we set the polarization of the pump photons to be elliptical with the fast axis in the vertical direction. The angles in the Jones vector (Equation 3) for this polarization are ϑ_*p*_ = 140° and φ_*p*_ = 90°. Polarization tomography is performed at different thicknesses of the sample to assess the polarization state and the degree of entanglement. The photon states are pure within the measurement error. The mean purity of twelve measured states at different thicknesses is 0.92 ± 0.10. Therefore, calculating the degree of entanglement based on measured density matrices from the concurrence as in Equation 2 is perfectly valid. The measured values are represented in black, while the red curve belongs to the simulated entanglement (Figure 2d). Thickness error was estimated to be 0.3 µm in each direction, based on the positioning accuracy and uncertainties of the LC cell thickness measurements. The concurrence values and their associated errors were determined from the mean and standard deviation of multiple shorter‐period measurements of the coincidence count histograms for each tomography setting. The same procedure was applied in all other measurements throughout the paper. The gray band contains the range of simulation results for ϑ = ϑ_*p*_ ± 5° and φ = φ_*p*_ ± 5°, which account for the estimated experimental uncertainty of pump polarization. We were able to generate almost completely entangled polarization states at 2.9 µm. At larger thicknesses, the concurrence gradually decreased as expected. Although the absolute numbers of the simulation do not exactly match with the measurements, the trend is nicely reproduced ‐ despite the simplifications in the model, such as using Jones calculus, having uncertainties in the material refractive indices, taking into account only collinear photons incident perpendicular to the LC surface and considering only generated photon pairs with central wavelength (double the wavelength of the pump photons). Nevertheless, we can tune the degree of entanglement by varying the sample thickness for a 180°‐twist director configuration. The difference in states at different thicknesses is additionally apparent from the density matrices of the generated photon pair states. Figure 2e,f represents real and imaginary parts of the density matrices at 2.9 µm and 11.4 µm, respectively. While there are several nonzero components in the density matrix for photons generated at 2.9 µm, there is only one significant component for polarization state at 11.4 µm, which comes from both photons having the same polarization and consequently leads to a lack of entanglement.

### 2.2 Electric Field Dependent Entanglement

**Figure 3:**
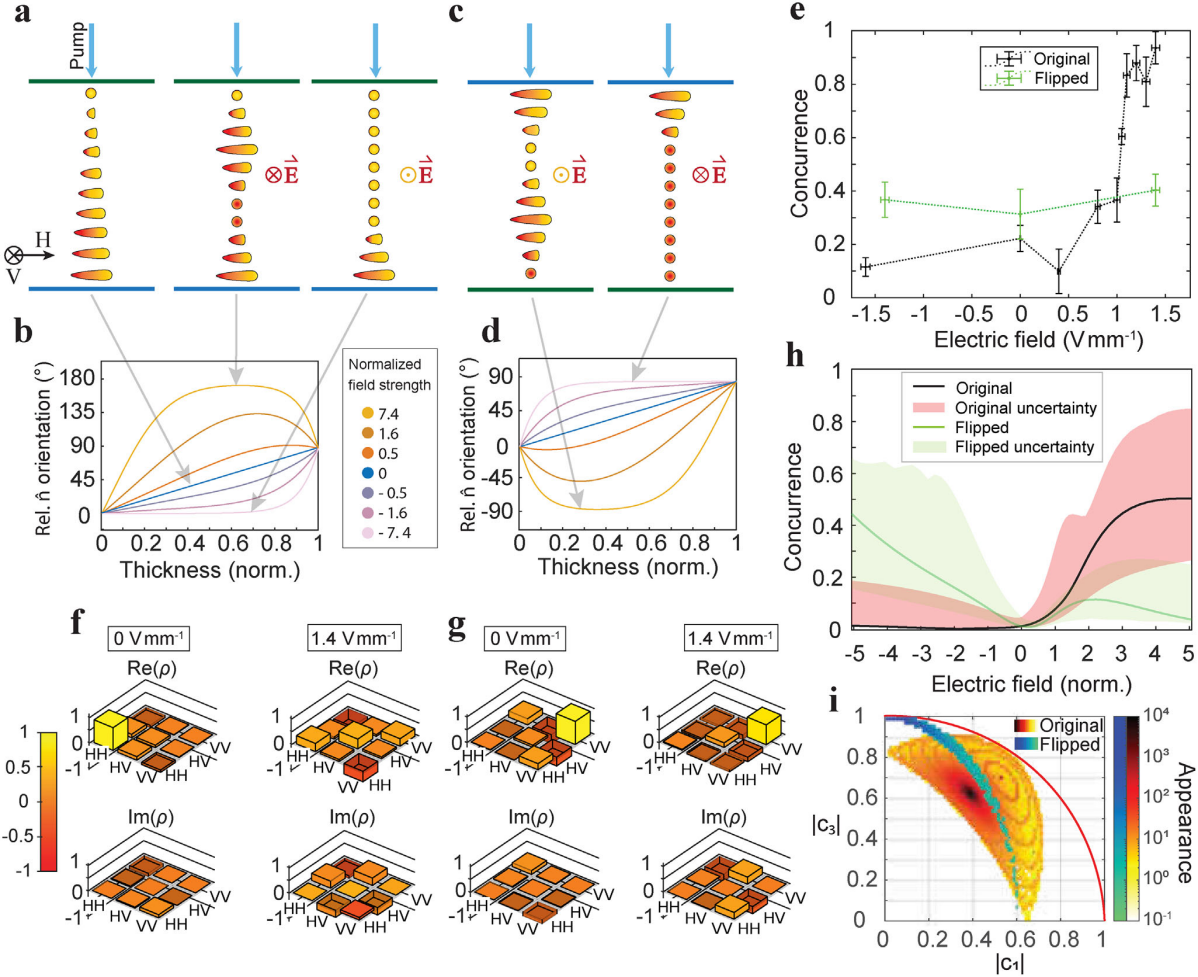
The influence of electric field on the director and consequently on the concurrence of generated photon pairs in the FNLC cell with right‐handed 90° twist. a) A schematic representation of the orientation of the director inside a cell with right‐handed 90° twist along the thickness. The top surface is rubbed parallel to the vertical direction and the bottom one parallel to the horizontal with respect to the pump beam polarization as defined in the figure. There is no electric field applied in the left subfigure, while there are electric fields present in the opposite directions in the middle and the right image. b) The simulated director orientation with respect to the orientation of the director at the top surface for a set of different electric fields. The blue curve represents the scenario where there is no electric field, while the yellow and pink curves correspond to three different strengths of positive and negative normalized electric fields, respectively. c) Schematic representation of the orientation of the director and d) simulated director profiles for the upside‐down flipped cell. e) Measured concurrence for different applied electric fields for the original (black points) and flipped (green points) orientation of the cell. f,g) Real and imaginary parts of density matrices for 0 V mm^−1^ and for 1.4 V mm^−1^ for original and flipped arrangement of the cell, respectively. h) Simulated concurrence as a function of the electric field strength for the original and flipped arrangement of the cell, respectively. The units of the electric field are normalized the same way as in (b,d). i) A histogram demonstrating relative appearance of different polarization states (plotted in terms of |*c*_1_| and |*c*_3_|) for the original and flipped arrangement of the cell, obtained from a simulation where the pump was varied across all possible polarizations. The red curve corresponds to the theoretical limit, where |*c*_1_|^2^ + |*c*_3_|^2^ = 1.

Next, we studied how the applied electric field influences the polarization state and concurrence. We performed measurements on a 5 µm thick cell with surfaces enforcing perpendicular orientations with respect to each other, so the director forms approximately a uniform right‐handed 90° twist along the sample if there is no field present (**Figure** **3**a). In terms of incoming pump polarization direction (or generated photon pairs), the top surface is rubbed in the vertical direction and the bottom one in the horizontal one, as defined in the image. When we apply an electric field between the electrodes, the director reorients within the gap (Figure 3a). The direction of rubbing prescribes the orientations of the director on the glass surfaces, while the interplay between elastic and electric interaction governs the orientation in the middle, creating a field dependent non‐monotonous twist. The larger the electric field, the larger the thickness range in which the FNLC polarization direction is parallel to it, with the fast twist confined close to the surfaces. The simulated azimuthal angular profiles of the director orientations are given in Figure 3b, where the angles correspond to the relative orientations of the director with respect to the orientation of the director at the top surface. The simulation is based on the dynamic analysis of the free energy, which includes elastic, electric, and surface energy terms. The electric field strength is normalized according to $E_{n}=10^{3}E_{0}d\sqrt{K_{2}\varepsilon_{0}}$, which is explained along with the simulation protocol in more detail in the Experimental Section. To analyse other possible polarization states that can be generated from the same cell and the same pump polarization, we also observed the upside‐down flipped cell, where the top and the bottom glass plates are switched (Figure 3c). The director orientation sequence is in this case inverted (Figure 3d).

The measurements were performed with a right‐circularly polarized pump beam, which, according to the simulation, should give higher concurrence in the presence of an electric field as opposed to a left‐circularly polarized light. The starting point of the measurements is the relaxed LC cell at 0V mm^−1^. The electric field is then gradually increased up to 1.4 V mm^−1^. The same procedure is repeated with a reversed electric field, where the field strength is increased up to ‐1.6 V mm^−1^. The value of electric field is determined from the width of the gap and applied voltage, with their estimated uncertainties of 50 µm and 2mV, respectively. We performed the polarization tomography at several electric field strengths in between and calculated the corresponding density matrices and concurrences. The values clearly show that although the concurrence is very low (*C* = 0.22 ± 0.05) at 0 V mm^−1^, we can controllably increase it by applying an electric field. When the electric field is increased to 1.4 V mm^−1^, we succeeded in getting almost completely entangled photons with concurrence 0.94 ± 0.06 (black points in Figure 3e). The reverse electric field yields lower concurrence (*C* = 0.12 ± 0.04) at ‐1.6 V mm^−1^. In the case of the flipped cell in the presence of an electric field, the director orientations from top to bottom are now different, as indicated by polarization profiles in Figure 3d. Unlike in the original orientation of the cell, the concurrence in this case remains below 0.5 for negative as well as positive applied electric field (green plot in Figure 3e). The generated states, therefore, in this case also depend on the orientation of the cell, because the pump beam experiences different twist structures in this two cases.

The difference between the states at different voltages and cell orientations can also be seen from the density matrices (Figure 3f,g). By far the largest component at 0 V mm^−1^ in original arrangement of the cell (Figure 3f) is the one corresponding to |2〉_*H*_|0〉_*V*_ state, which means the majority of photon pairs are horizontally polarized and therefore non‐entangled. With increasing electric field, the generation of vertically polarized photons increases, which leads to an increasing presence of additional components of the density matrix (Figure 3f). For the flipped cell, the density matrix component for both photons being vertically polarized remains large for both zero and non zero (*E* = 1.4V mm^−1^, Figure 3g) applied electric field, leading to low entanglement.

To compare the measurements with the theory, we simulated the concurrence of generated photon pairs for both the original and the flipped arrangement of the cell. Unlike the simulations performed in our previous work,^[^ ^13^ ^]^ where we were only able to simulate SPDC from a uniformly twisted cell, we have now implemented the possibility to calculate the state and intensity in a sample with an arbitrary director profile, where the pitch is long enough so that the Jones formalism still gives results to a sufficient degree of accuracy. This new approach enables the simulation of the photon pair generation in the presence of an electric field. A more detailed explanation of the simulation can be found in the Experimental Section. Results of the simulation for original and flipped orientation of the cell are shown in Figure 3h. The simulation predicts an increase in concurrence with increasing electric field in the positive direction and low concurrence in the presence of the field in the opposite direction for the original arrangement of the cell. It turns out that the simulation strongly depends on the values of refractive indices, which were calculated using the formula *n* = *A* + *B*/λ^2^, where coefficients *A* and *B* were obtained from the measurements carried out in our previous work.^[^ ^13^ ^]^ The uncertainty concurrence band (red in Figure 3h) encompasses 5% variation in *A* for extraordinary refractive indices, which is reasonable according to our recent paper.^[^ ^13^ ^]^ With this slight variation, the concurrence uncertainty band extends to values of more than 0.8 at high electric fields. The same variation in extraordinary refractive indices is used for the uncertainty band in the flipped arrangement of the cell (green in Figure 3h). The other possible origins of discrepancies between the measurements and the simulations are fixed wavelength (900 nm) of the generated photon pairs, neglect of different outgoing photon angles, and uncertainties in the electric field profiles, together with the invalid Jones formalism for fast twists. However, the simulation still predicts the same qualitative response of the electric field on concurrence as measured. In the case of traditional sources of entangled photons, such as two orthogonally oriented nonlinear crystals,^[^ ^35^ ^]^ the geometry is simple and we can easily understand why a particular geometry creates entanglement. In our case, however, the situation is very complex, since our system does not originate from a single dominant feature of the twisted FNLC cell, but rather from the combined action of several parameters—including cell thickness, twist angle, birefringence, nonlinear d‐tensor elements and pump polarization. Only under certain conditions do these contributions interfere in a way that leads to high degrees of entanglement, while other scenarios lead to a broad spectrum of different states. Consequently, the origin of entanglement cannot be easily described by an intuitive explanation. However, although the entanglement in FNLCs depends on multiple parameters, including the applied electric field, which adds yet another layer of complexity by significantly modifying the director profile, there is a clear overall trend in concurrence when applying an electric field. Although fine features are indeed complex due to the many coupled parameters, the overall trends remain intuitive and consistent between theory and experiment.

The measurements, as well as the simulation, confirm that the generated states differ for the original and flipped orientations of the cell. To see if there is also a difference in the number of possible states that any polarization can generate, we simulated all different pump polarizations (angles ϑ_*p*_ and φ_*p*_ in Jones vector were varied in steps of 1°) and present the number of certain generated states in a histogram (Figure 3i). The states generated in a flipped cell cover a different region than the states from the original orientation of the cell, which means the possibility of flipping the cell broadens the spectrum of states, that can be generated. The applied electric field breaks the symmetry of the produced states when the sample is pumped in either forward or backward directions.

**Figure 4:**
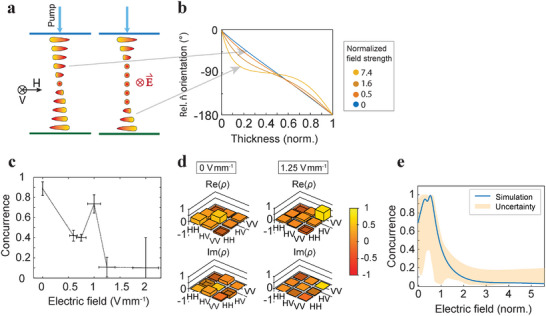
The influence of electric field on the director in the sample and consequently on the concurrence of generated photon pairs in the FNLC cell with 180 ° twist. a) FNLC cell with 180 ° director twist along its thickness without external electric field and in the presence of electric field. b) Simulated director profiles for different applied electric fields. The electric field strength is normalized the same way as in Figures 3b,d. c) Measured values of concurrence at different external electric field values. d) Real and imaginary parts of density matrices for generated photon pairs at 0V mm^−1^ and at 1.25 V mm^−1^. e) Simulated concurrence as a function of the electric field strength.

Above we have shown that we can increase the concurrence from approximately *C* = 0.3 to more than *C* = 0.9 with the presence of an electric field in a cell with a 90° twist. To show the opposite effect, namely to decrease the concurrence with electric field, we have assembled a 3.5 µm thick cell with antiparallel rubbing on the plates, so that 180° twist of the director is induced in the absence of an electric field (**Figure** **4**a). We again use the pump polarization characterized with ϑ_*p*_ = 140° and φ_*p*_ = 90° and perform the measurements in the left‐handed domain with applying the electric field in the direction that deforms the structure toward uniform orientation without nucleating opposite domains.^[^ ^33^ ^]^ The director profiles are simulated in Figure 4b. The obtained measurement results are shown in Figure 4c. The concurrence decreases from *C* = 0.89 ± 0.07 at 0V mm^−1^ to *C* = 0.1 ± 0.1 at 1.25V mm^−1^, which means we have achieved the decrease in concurrence with electric field. The initial director configuration and high concurrence can be restored by turning the field off. The polarization states are represented with density matrices in Figure 4d. For 1.25V mm^−1^, there is only one prevailing component in the density matrix, which indicates low photon entanglement. The simulation (Figure 4e) is done the same way as for 90° cell. The uncertainty band is again obtained with 5% change in extraordinary refractive indices, to which an additional 2 ° change in incoming light polarization is added as well. Although the peak in concurrence is shifted a bit to the positive values of the electric field, the decrease in concurrence with increasing electric field is still clearly visible.

## 3 Conclusion

In conclusion, we have successfully demonstrated that FNLC can be used as a tunable source of entangled photon pairs. Gradual change in the degree of entanglement can be achieved by constructing an LC cell with increasing thickness, the right twist of the director and suitable incoming beam polarization. We succeeded in generating photon pairs with concurrence that ranged from 0.98 ± 0.06 to 0.076 ± 0.11, depending on the position on the cell. With this, we also for the first time demonstrated the high degree of entanglement from an LC source. Secondly, we accomplished tuning the degree of entanglement by exposing the FNLC to an external electric field. We proved that the concurrence can be either decreased or increased with the electric field, depending on the pump beam polarization, construction of the FNLC cell and its orientation. Moreover, if the electric field is present in 90° twist cell, the number of possible generated states increases, because the original and the flipped orientation of the cell produce different polarization states. Tuning the degree of entanglement with the electric field is a reversible and technically easily achievable method and is, as such, a promising method for future quantum applications, e.g., quantum key distribution, quantum information processing and possible development of multipixel tunable quantum devices. In the latter case, the electric field in each pixel could be individually controlled to obtain the desired pattern of photon pairs with a varying degree of entanglement. Overall, we believe that tunability of polarization entanglement both with thickness and with electric field is an important step in the development of tunable quantum optic devices.

## 4 Experimental Section

### Material and Cell Preparation

The material used in this study was the ferroelectric nematic liquid crystal FNLC‐1751, provided by Merck Electronics KGaA. Its phase transition temperatures on cooling from isotropic phase were: isotropic to non‐polar nematic at 87°, nematic to antiferroelectric modulated nematic^[^ ^4^, ^36^, ^37^ ^]^ at 75 °C and finally, ferroelectric nematic phase at 45 °C, which was stable down to room temperature. The material possesses second‐order nonlinear properties with the only significantly large component of *d* tensor being *d*_33_ = 20pm V^−1^.^[^ ^13^ ^]^

Liquid crystal cells were prepared from fused silica glass to avoid laser‐induced fluorescence. Glasses meant for electrodes were coated with transparent indium tin oxide (ITO) and etched to create a 500 µm wide gap without ITO from one edge of the cell to the other. The two electrodes were mounted in such a way that the applied electric field caused an in‐plane field in the gap region. A 20nm thin layer of 30% solution of polyimide SUNEVER 5291 (Nissan) was coated on top of all glasses and then rubbed along the gap or, in case of no gap, along one of the edges of the cell to ensure the in‐plane anchoring of the molecules at the surfaces along that direction. The two glasses were then glued together, so the rubbing directions were either anti‐parallel or perpendicular to each other. The twist of the director along the sample was consequently 180° or 90°, respectively. Plastic beads (EPOSTAR) were used as spacers to obtain the desired cell thickness. The cells were either of constant thickness or wedge‐shaped, whereby their thicknesses ranged between 2.5 µm and 20 µm. The cell sizes were around 1 × 1cm^2^.

Liquid crystal was injected into the cell in the isotropic phase at 100 °C by capillary forces. The sample was then cooled down to room temperature at 1°C/min and was left to stabilize itself outside the heating stage before measurements.

### Measurements

For all these measurements, a continuous‐wave (CW) laser was used with the central wavelength of 450 nm and 1mW power to serve as a pump beam (Figure 1a). Before sending the beam to the objective, its polarization was adjusted with a polarizer (POL), a half‐wave plate (HWP), and a quarter‐wave plate (QWP). To focus the light on the sample, a 10 × objective with a numerical aperture of 0.3 was used (OBJ1). The outgoing light was sent to a 20 × objective with a numerical aperture of 0.5 (OBJ2). Two long‐pass filters were used with cut‐off wavelengths at 496 nm (LP1) and 700 nm (LP2), respectively, to eliminate the laser light. Their optical density was, according to specifications, greater than 4 and 5. When performing measurements in the presence of an electric field, the sample electrodes are connected to a function generator. The last part of the optical path was a Hanbury Brown ‐ Twiss ‐ like setup with a 50:50 non‐polarizing beam splitter (NPBS). Each arm contains a half‐wave and a quarter‐wave plate to rotate the desired light polarization to horizontal. A polarizing beam splitter (PBS) was used as a polarizer to allow only horizontally polarized light to pass through. An additional lens in each arm was used to focus the beam and another long‐pass filter (LP3) with a transmission cut‐off at 840 nm to eliminate as much background as possible. The light was detected with two equal detectors, SPCM‐AQRH Single Photon Counting Module from Excelitas Technologies (DET1 and DET2). They were avalanche photo diode‐based, with a 70 Hz dark count rate and bin time resolution of 100 ps.

### Tomography Protocol

Count rate of photon pairs, created via SPDC, was obtained from the histogram of the time delays between two consecutively detected events on the detectors. The count rate corresponds to the area under the peak of the histogram at time delay 0 s with subtracted background count rate. Because we are interested in not only the count rate but also the polarization state of these photons, a polarization tomography was performed. The base for calculations consists of Fock states |2〉_*H*_|0〉_*V*_, |1〉_*H*_|1〉_*V*_ and |0〉_*H*_|2〉_*V*_, where *H* corresponds to horizontal polarization and *V* to the vertical one. Therefore, the state can be described with a 3 × 3 density matrix $\hat{\rho }$, where $\rho_{nm}=\rho_{mn}^{*}$. Consequently, we need to find 9 independent real numbers to construct the density matrix. To do this, we measured 9 different combinations of photon pair polarizations. In particular, we choose to measure count rates of photon pairs with polarizations *HH*, *HV*, *VV*, *HD*, *HR*, *VA*, *VL*, *DD* and *DR*, where *D* stands for diagonal, *A* anti‐diagonal, *R* right hand circular and *L* left hand circular polarization. Each combination of polarizations corresponds to certain angles of lambda half and quarter wave plates in front of the detectors.

The states we generate are, to a good approximation, pure, so the absolute values of complex coefficients *c*_1_, *c*_2_, and *c*_3_ of a wave function |ψ〉 = *c*_1_|2〉_*H*_|0〉_*V*_ + *c*_2_|1〉_*H*_|1〉_*V*_ + *c*_3_|0〉_*H*_|2〉_*V*_ are equal to the square roots of the diagonal elements of the matrix. We can define the number operator for photon pairs with polarizations *i* and *j* as ${\hat{n}}_{ij}=\left\langle {\hat{a}}_{i}^{†}{\hat{a}}_{j}^{†}{\hat{a}}_{i}{\hat{a}}_{j}\right\rangle$, where ${\hat{a}}^{†}$ and $\hat{a}$ are creation and annihilation photon operator, respectively. Its expectation value can be written as

(4) $$\left\langle {\hat{n}}_{ij}\right\rangle =\mathrm{Tr}\left(\hat{\rho }{\hat{n}}_{ij}\right),$$

where Tr stands for matrix trace. The expression equals to

(5) $$\begin{array}{ccc} \left\langle {\hat{n}}_{ij}\right\rangle & = & {\left\langle 2\right|}_{H}{\left\langle 0\right|}_{V}\;\hat{\rho }{\hat{n}}_{ij}{\;|2\rangle }_{H}{\left|0\right\rangle }_{V}+{\left\langle 1\right|}_{H}{\left\langle 1\right|}_{V}\;\hat{\rho }{\hat{n}}_{ij}{\;|1\rangle }_{H}{\left|1\right\rangle }_{V}\\ & & +{\;\langle 0|}_{H}{\left\langle 2\right|}_{V}\;\hat{\rho }{\hat{n}}_{ij}{\;|0\rangle }_{H}{\left|2\right\rangle }_{V}. \end{array}$$

### Simulations

The simulations were performed by using 1D Jones matrix formalism to calculate the polarization state of the photon‐pairs generated from an FNLC with *d*_33_ as the only non‐zero component of the *d*‐tensor and refractive indices according to the measured values. The polarization of the pump at each position along the sample was calculated. Based on this, as well as the local orientation of the molecules, the polarization and intensity of the outgoing photon pair were calculated. This newly generated pair was propagated to the end of the sample. At the end the contributions from all of the points along the thickness were summed to obtain the polarization state wavefunction. More details regarding the calculation protocol can be found in ref. [13].

Additionally, the simulations implemented within this work enable the calculation of a two‐photon polarization state of an arbitrary twisted structure. For this purpose, the sample was divided into 20 layers per micrometer with known orientations of the director. Jones matrix formalism was used for each transition from one layer to another. The matrices from each layer were multiplied together to obtain the whole polarization transformation during propagation of the photons. This means we can move from a perfect linear twist profile to a twist changing at different rates along the thickness of the liquid crystal cell. This becomes useful when trying to estimate the effect of the applied electric field on the two‐photon state. As the field was applied, it rotates the molecules away from the initial linear profile. The new twist profile was calculated according to the free energy density described in detail in reference,^[^ ^38^ ^]^ where polarization was considered parallel to the director, and the coupling of the applied electric field with the polarization was taken linear (− **P** · **E**) and the different twist profiles ±90° or ±180° are considered. In the present case, the following parameters were considered: cell thickness d=5μm, P=5μ C/cm^2^,^[^ ^39^ ^]^ *K*_2_ = 0.2pN,^[^ ^40^ ^]^ $\eta_{n}=0.1\mathrm{Pa}s$ ^[^ ^36^ ^]^ and strong anchoring conditions *W* = 1 × 10^−4^ J/m^2^. The final equilibrium structure is then calculated by solving the dynamic equation as described in reference.^[^ ^38^ ^]^ It should be noted that such a model was simplified and neglects internal field effects, which, given the material's spontaneous polarization values, are expected to be large. Thus, nominal normalized electric fields for simulations ($E_{n}=10^{3}E_{0}d/\sqrt{K_{2}\varepsilon_{0}}$) differ from experimental ones. The factor 10^3^ is introduced to remove leading zeros in the graph representation.

## Conflict of Interest

The authors declare no conflict of interest.

## Author Contributions

S.K. and A.K. performed the experimental work, theoretical simulations, and analysis of the results. N.S. helped with sample preparation and the theoretical simulations of field induced structures. M.H. conceived the original idea and supervised the study. All authors wrote the manuscript and approved the final version of the paper.

## Data Availability

The data that support the findings of this study are available from the corresponding author upon reasonable request.
